# Effects of physical exercise on central nervous system functions: a review of brain region specific adaptations

**DOI:** 10.1186/s40303-015-0010-8

**Published:** 2015-04-18

**Authors:** Julie A Morgan, Frances Corrigan, Bernhard T Baune

**Affiliations:** University of Adelaide, School of Medicine, Discipline of Psychiatry, Psychiatric Neuroscience Laboratory, Adelaide, South Australia Australia; University of Adelaide, Discipline of Anatomy and Pathology, School of Medical Sciences, Adelaide, South Australia Australia

**Keywords:** Exercise, Neurophysiology, Neurobiology, Brain stem, Hypothalamus, Basal nuclei, Disease, Depression, Stress, Neurodegenerative Diseases

## Abstract

Pathologies of central nervous system (CNS) functions are involved in prevalent conditions such as Alzheimer’s disease, depression, and Parkinson’s disease. Notable pathologies include dysfunctions of circadian rhythm, central metabolism, cardiovascular function, central stress responses, and movement mediated by the basal ganglia. Although evidence suggests exercise may benefit these conditions, the neurobiological mechanisms of exercise in specific brain regions involved in these important CNS functions have yet to be clarified. Here we review murine evidence about the effects of exercise on discrete brain regions involved in important CNS functions. Exercise effects on circadian rhythm, central metabolism, cardiovascular function, stress responses in the brain stem and hypothalamic pituitary axis, and movement are examined. The databases Pubmed, Web of Science, and Embase were searched for articles investigating regional brain adaptations to exercise. Brain regions examined included the brain stem, hypothalamus, and basal ganglia. We found evidence of multiple regional adaptations to both forced and voluntary exercise. Exercise can induce molecular adaptations in neuronal function in many instances. Taken together, these findings suggest that the regional physiological adaptations that occur with exercise could constitute a promising field for elucidating molecular and cellular mechanisms of recovery in psychiatric and neurological health conditions.

## Review

### Introduction

Health can be disrupted by stress of acute or chronic duration, and may be either physiological or psychological [1]. Extreme stressors that elicit the acute ‘fight or flight’ responses, such as violence or natural disasters can and do occur however, the chronic low level daily hassles or issues that cause stress and result in sleep loss, comfort eating with resultant weight gain, and smoking or excessive drinking of alcohol are more common [2]. Chronic stress is considered to contribute to the aetiology of a range of psychiatric and neurological conditions including depression and Alzheimer’s disease [3,4]. Furthermore, these conditions often involve the dysregulation of important functions coordinated by the brain such as circadian rhythms [5,6], central metabolic function [7,8], and stress responses via the hypothalamic pituitary adrenal axis (HPA) [9,10].

Basic science and clinical research is providing promising evidence of physical exercise-induced outcomes for several prevalent neurological and psychiatric conditions (CNS). This occurs in part through increases in neurotrophic factors such as brain derived neurotrophic factor (BDNF) [3,11,12], reductions in oxidative stress [13] and limiting neuroinflammation [14,15]. However many of the mechanisms by which exercise exerts its effects in the brain remain largely unknown.

Nevertheless, a substantial body of literature has now investigated the effects of exercise in a range of populations [16-18], and brain regions such as the hippocampus [19-21], resulting in advancement in the understanding of the exercise on a number of areas including cognitive functioning and the neurobiology of learning and memory. However, considerably less work has investigated the impacts of exercise on more primitive brain regions including the brainstem, hypothalamus, and basal ganglia, which are involved in other important functions for health. These include the regulation of diurnal rhythm and circadian function, food intake, cardiovascular function, and responses to stressors. There is increasing recognition of metabolic dysfunction in Alzheimer’s disease [22] and depression [23]. Moreover, there is growing evidence that brain metabolic disturbances such as central insulin resistance are involved in the pathogenesis and progression of Alzheimer’s disease [22], and that circadian rhythm and HPA axis disturbances can be evident in depression and Alzheimer’s disease [5,6,10]. Given the roles of these CNS dysfunctions in the aetiology and progression of these conditions, understanding the regional neurobiology of such mechanisms seems critical for advancing preventative measures and treatments. The aim of this review is therefore to elucidate and critically evaluate the effects of chronic exercise in the context of basic drive functions in the brainstem, hypothalamus, pituitary gland and basal ganglia. Particular focus will be on the exercise-induced regulatory effects on energy balance and metabolism, cardiovascular regulation, circadian function, and responses to stress.

## Materials and methods

The PRISMA guidelines (Preferred Reporting Items for Systematic Reviews and Meta-analysis) for reporting systematic reviews and meta-analyses checklist items were followed in the reporting of this review (for the items eligibility criteria; information sources; search; and study selection) [24].

### Searches

**Figure 1:**
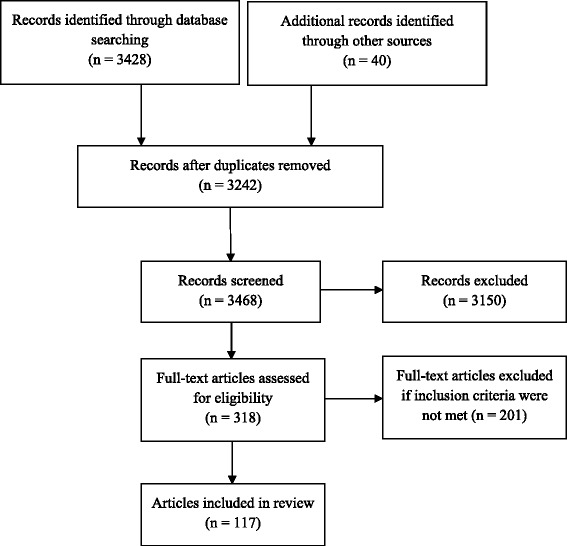
Flow diagram of included studies (adapted from [24]).

Searches were conducted in the electronic databases Pubmed, Embase, Medline, and Web of Science. The search terms exercise; voluntary wheel running, and wheel running were combined using OR, then combined using AND with the terms: brain stem; hypothalamus; paraventricular nucleus; suprachiasmic nucleus; ventromedial nucleus; thalamus; basal nuclei; neurobiology; energy; metabolism; metabolic; autophagy; circadian; diurnal; cardiovascular; sympathetic; parasympathetic; and HPA axis. The 3408 articles returned were screened by review of the titles and abstracts for relevance to the aims of this paper, and contained 222 duplicates. Papers were exported and stored in Endnote X6.0.1 software for further consideration of the full text (see Figure 1).

### Inclusion and exclusion criteria

Articles published in the English language were selected involving murine studies on adult animals. Murine studies investigating chronic exercise-induced effects on central neurobiological functions in the brain stem, hypothalamus, thalamus, and basal ganglia were included. Given that the focus of this review relates to murine neurobiological mechanisms in the brain stem, hypothalamus, thalamus, and basal ganglia, research investigating human participants was excluded. In addition, murine studies investigating the effects of exercise on peripheral and plasma measures; drug interventions; addiction; post traumatic brain injury or spinal cord injury; sexual function and dysfunction; autism spectrum disorders; attention deficit hyperactivity disorder; gene expression; and whole brain analyses without regional brain distinctions were excluded. Other brain regions including those related to the limbic system such as the hippocampus; cortex; amygdala; and prefrontal cortex were excluded because these regions are involved in emotion and cognition generation rather than fundamental physiological processes. Although exercise-induced physiological interactions between the limbic system and the brain stem, hypothalamus, thalamus, and basal ganglia require investigation, these topics are complex and will require extensive investigation that is beyond the scope of this review. Fifty-seven papers without full text were excluded, and searches were limited to the years 1998 to 2015. The final databases searches were conducted on 3rd February 2015.

## Results and discussion

### Voluntary and forced exercise methods

Several noteworthy points about murine research involving exercising animals require clarification. Murine studies investigating adaptations to exercise utilise a range of methodologies involving varied types of exercise, such as voluntary wheel running (VWR) or forced exercise. They also utilise different intensities of exercise ranging from low intensity, moderate, or high intensity. Mice running voluntarily on a running wheel tend to run intermittently in short bursts and at a preferred cruising speed [25]. However, forced exercise often involves speeds set at a constant rate, for example on a rodent treadmill set at 8 metres/minute, or incrementally increased speeds over the duration of the exercise program. In addition, forced exercise is believed to involve the potential additional component of emotional stress from coercion of the animal, and this makes it difficult to differentiate between the effects of the physical stress of exercise, and the effects of the emotional stress of coercion, thereby potentially confounding the investigated outcomes [12,26] (see Table 1). The voluntary or forced mode of exercise undertaken by animals is therefore an important concern, and is reported throughout this review.

**Table 1:** **The differences between voluntary and forced exercise in murine studies**

**Voluntary exercise**	**Forced exercise**
Self-selected cruising speed	Researcher pre-prescribed speeds
Variable speeds	Constant speeds
Short bursts of exercise	Relatively long periods of exercise
No coercion of the animal	Coercion of the animal
No psychological stress arising from coercion	Possible psychological stress arising from coercion
No physiological cascades arising from psychological stress from coercion	Possible physiological cascades arising from potential psychological stress from coercion
No potential for physiological responses to the psychological stress of coercion to interact with or confound the parameters under investigation	Potential for physiological responses to the psychological stress of coercion to interact with or confound the parameters under investigation
No potential for confounded findings due to psychological stress physiology	Potential for confounded findings due to physiological stress physiology
Directly translatable to clinical studies	Difficulty with translation to clinical studies

## Brain stem

### Dorsal raphe nucleus adaptations to exercise

The dorsal raphe nucleus contains serotonergic neurons that have extensive projections to many brain regions. These include those involved with mood states and behaviour [27], such as the amygdala, hippocampus and cerebral cortex [28] that are widely implicated in stress, anxiety and depression [29]. VWR has a range of impacts on serotonin-mediated responses to stressors. These include effects on serotonin receptors that when activated, inhibit serotonin synthesis and release, and are thus implicated in resilience to stress and anxiety [30]. Six weeks of VWR reduced the activation of serotonergic neurons in the rostral and mid dorsal and ventral DRN in response to uncontrollable stress, as detected by *c-fos* staining [31]. This may be a mechanism that contributes to reducing stress responses in rats.

There are also time dependent changes in the receptor mRNA in the DRN. Three weeks or six weeks, but not 3 days of VWR, increased the mean serotonin transporter (5HTT) mRNA (conducts the reuptake of extracellular serotonin into presynaptic neurons) in the DRN (p = 0.02) [32]. The mean DRN serotonin receptor 1A (5HT_1A_) mRNA (p = 0.05) was also increased [32]. In addition, 3 days, 3 weeks and 6 weeks VWR decreased serotonin receptor 1B (5HT_1B_) mRNA in the rostral and mid ventral DRN [32]. Interestingly, transient increases in mean α_1*b*_*-*adrenergic receptor (α_1*b*-_ADR) mRNA at three weeks had returned to baseline levels at 6 weeks [32]. The temporal aspects of these changes suggest that the duration of VWR is a factor effecting 5HTT, 5HT_1A_ mRNA, 5HT_1B_ mRNA, and α_1*b*-_ADR mRNA in the DRN [33]. Importantly, VWR appears to induce mechanisms that directly affect serotonergic neuron excitability and inhibition in the DRN.

Time dependent adaptations with VWR are also evident in stress induced behavioural parameters mediated by the DRN [34,35]. VWR induced reductions in uncontrolled stress exacerbated behavioural deficits in shuttle box escape latencies were evident at 6 weeks but not at 2 weeks [34]. Curiously, 6 weeks of forced wheel running and VWR, but not forced treadmill running were found effective in reducing uncontrollable stress induced deficits in learning [35]. This suggests that exercise involving a forced component may be therapeutic in some instances [35]. In summary, VWR results in time dependent changes in basal levels of 5HTT, autoreceptor 5HT_1A_ and 5HT_1B_ mRNA, and α_1*b*-_ADR in the DRN in region specific ways. These factors appear to be involved in VWR induced attenuations in uncontrollable stress induced deficits in latencies to escape from shuttle box testing in rats. The effects of exercise on serotonin modulation in the DRN are therefore noteworthy for their positive effects on behavioural responses to stress. Speculatively speaking, if serotonergic modulation occurs in the DRN with VWR, this could mediate the input of serotonergic neurons to regions such as the amygdala and hippocampus and have subsequent effects on limbic and cognitive functions. Moreover, the modulation of serotonin in the DRN [31] also has potentially important implications for serotonergic afferent neuronal pathways linking the suprachiasmic nucleus in the hypothalamus that are involved in circadian rhythm function, and this is addressed later in the review.

### Exercise-induced changes in the locus coeruleus

Noradrenergic neurons in the locus coeruleus (LC) are involved in the regulation of attention, arousal, and vigilance responses to stress [36,37]. Stress responses arising from the LC occur in part through signalling via norepinephrine accompanied by galanin - a regulatory peptide formed from the cleavage of preprogalanin. Galanin attenuates neuronal hyper-excitability and may therefore be involved in the noradrenergic neurons adaptation to stress [38]. Exercise induces a range of effects on galanin and preprogalanin. VWR for 5–6 weeks reduced noradrenalin during and after foot shock stress, although it did not alter mRNA expression of TH or levels of galanin in the LC [39]. In contrast, three weeks of VWR resulted in significant elevations of galanin in the LC after contextual fear conditioning [40]. The authors attributed this to being due to dose dependent differences in the distances run, with their Long-Evans rats running around 20 times further [40] than the Fischer 344 rats [39]. Later studies attempted to control for the confounding factor of the stressors involved in the fear conditioning paradigm by removing stressful behavioural testing, and found that 3 weeks of VWR increased both preprogalanin and galanin expression in the LC [37,41]. Moreover, increased galanin mRNA has also been demonstrated after 3 weeks of VWR in rats selectively bred for greater aerobic capacity, with a correlation evident between the distance run and galanin expression (r – 0.317, p – 0.028) [42]. Voluntary exercise therefore appears to increase galanin and preprogalanin, with possible correlations between its expression and the distances run. Of particular note, is that the locus coeruleus has excitatory input into activation of the hypothalamic pituitary axis (HPA) responses to acute stressors [43]. Elevations in galanin and preprogalanin could therefore contribute to attenuating locus coeruleus excitatory responses to acute stress, and this may have effects on downstream HPA activation. Further investigation is required to clarify this possibility.

## Hypothalamus

### Circadian clock adaptations to exercise

The hypothalamus has an instrumental role in coordinating visceral and drive functions. Functions of the hypothalamus include maintaining energy balance, metabolism, autonomic nervous system modulation, and the circadian clock. The circadian clock is a timing mechanism that endogenously coordinates biochemical, physiological, and behavioural processes with the 24 hour cycle of light and dark [44]. Circadian functioning deteriorates with ageing, and can be disrupted by chronic stress [45]. Circadian dysfunction is implicated in the progression of neurodegenerative conditions [46] and in depression, possibly through alterations in hormones such as cortisol, norepinephrine and melatonin [6].

There is increasing evidence that exercise has noteworthy effects on sleep/wake cycles and circadian clock modulation in both humans and rodents, although the mechanisms involved are not fully understood. The suprachiasmic nucleus (SCN) is considered to be the central coordinating nucleus of circadian functioning although this occurs with some involvement of the brain stem [47]. In rodents, vigorous voluntary wheel running *ad libitum* provides feedback to regulate the central circadian clock and scheduled exercise can contribute to entraining circadian behaviour [48]. These adaptations occur in part via VWR related arousal signals that relay from dorsal raphe serotonergic pathways to the SCN [48,49]. The voluntary and spontaneous movement of mice such as grooming, moving or walking, acutely modulates SCN circadian clock pacemaker activity by reducing the amplitude of SCN electrical activity in a duration and intensity-dependant manner [47]. This is consistent with other findings that age related declines in SCN amplitude and rhythmicity in male mice are attenuated with access to a running wheel [50]. VWR increases serotonin in the SCN suggesting that serotonin could be a mechanism that mediates SCN amplitude [49]. Indeed, selective lesion of 5-HT terminals in the SCN prevents VWR induced circadian synchronicity [49]. Finally, VWR induces adaptations in hypothalamic heat shock proteins. Heat shock proteins are families of proteins that have robust cytoprotective properties and act as chaperones for other intracellular protein molecules, thereby contributing to cellular resistance to stressors [51]. VWR for 6 weeks in adult male Fischer rats induced elevated levels of the heat shock protein72 (HSP72) (p = 0.0019) in the context of interleukin 1 beta immune challenge in the hypothalamus, suggesting that VWR induces greater cellular resistance to immune challenge stress in this region [52]. Findings from rodent studies are consistent with results from human trials, and together suggest that exercise has potential for altering aspects of circadian dysfunction [48]. This has encouraging potential for a range of human conditions because poor sleep is thought to be a factor in the aetiology of prevalent mental health conditions including anxiety and depression [53], and may also be involved in the pathophysiology of neurodegenerative conditions such as Alzheimer’s disease [5].

### Exercise effects food intake and energy balance

The hypothalamus also contains nuclei involved in maintaining energy balance, including the arcuate nucleus, paraventricular nucleus, and dorsomedial and ventromedial hypothalamus. Energy intake and imbalance are contributing factors in the aetiology of neurodegeneration. More specifically, insulin resistance and diabetes in midlife is a risk factor for Parkinson’s disease in later life [54,55], and a poor diet involving high fat intake or metabolic disruption such as metabolic syndrome (MetS) can contribute to disease progression in Alzheimer’s disease [7,8]. Healthy leptin and insulin signalling in the hypothalamus are central factors in energy balance mechanisms, because reductions in these cascades can result in an increase in food intake and weight gain or obesity [56-58].

Short term VWR impacts on various CNS measures related to energy balance and food intake. VWR for 2 days to 1 week reduces the intake of high-fat chow in Sprague–Dawley and F344 rats [59,60]. This occurs via reduced meal size and meal frequency from activation of the corticotropin-releasing factor (CRF/CRH) pathway in the dorsomedullary hypothalamus [59]. A significant increase in leptin signalling in the ventral tegmental area also occurs [60], and these factors suggest VWR has a modulatory effect on food choice through CRF and leptin signalling [59,60]. Furthermore, the delivery of an exogenous leptin receptor antagonist in the hypothalamus results in significantly reduced VWR (p = 0.03) in F344-Brown Norway rats [61]. However, forced exercise also has metabolic effects in the hypothalamus. Forced uphill treadmill running for 30 minutes, 4 times weekly, for 8 weeks increased tyrosine phosphorylation of insulin receptor 2 (IRS2) with corresponding elevations in IRS2 and subsequent increases in Akt phosphorylation and insulin signalling in the hypothalamus [58]. However, VWR was not included in this study to ascertain potential differential effects. It is possible that the stress from coercion resulted in altered neurophysiological metabolic responses to exercise, thereby confounding the results. Nevertheless, both VWR and forced exercise may result in beneficial alterations to central metabolic status.

The effects of longer term VWR on weight and metabolic status at different stages of the lifespan were investigated in the Berlin Fat Mouse Inbred stain - bred for its predisposition for the development of obesity and metabolic syndrome. Chronic VWR in this breed resulted in the amelioration of weight gain, body fat mass, daily energy intake, and peripheral features of MetS arising from a high fat diet [62]. Similarly, in a Sprague–Dawley model of mid-older age obesity, 2 weeks of VWR inhibited food intake (nearly 50% or p < 0.001) [63]. These reductions occurred with modest amounts of VWR that significantly increased leptin signalling in the ventral tegmental area (VTA) (but not the hypothalamus) with resultant reductions in high fat-diet intake and subsequent weight loss [63]. It is noteworthy that more recent work utilising forced methods suggests that chronic forced treadmill exercise does not sensitise leptin function in the hypothalamus [64]. Further work is therefore required to clarify this inconsistency and the mechanisms involved, and in particular, whether physiological cascades involved with a stress response to forced exercise are involved. Interestingly, starting VWR prior to adulthood (at 3 weeks of age) resulted in reduced food intake, whilst initiating exercise from early adulthood (at 9 weeks) increased food intake, although circulating insulin levels remained within the normal range [62]. VWR may therefore attenuate characteristics of Mets arising from diet related energy imbalances and obesity, and may have age related effects on food intake. Further studies on these topics would provide useful clarification about these factors.

Exercise induces autophagy in peripheral skeletal muscle and cardiac tissue, and is a mechanism that contributes to exercise-induced glucose homeostasis via the BLC2 phosphorylation sites [65]. Autophagy is characterised by lysosomal degradation pathways that transfer materials from the cytoplasm to the lysosome. This serves to recycle cellular components such as damaged organelles and aggregated proteins for cellular nutrition during starvation, or to meet higher energy demands [66,67]. Although studies investigating potential exercise-induced autophagy in brain regions has not identified its occurrence in the hypothalamus, exercise increases the transgenic fluorescing autophagy marker GFP-LC3 in the anterior cerebral cortex [68]. The potential for autophagy in the hypothalamus and other brain regions requires further careful investigation, because the authors noted the possibility that different methods of sample preparation might result in more sensitive detection of autophagy markers in other brain regions [67]. This is an important topic for investigation because disruptions of autophagy are implicated in neurodegenerative conditions [68]. Future examinations of exercise-induced autophagy in other brain regions therefore ought to utilise methods that are more sensitive so that the mechanisms involved could be elucidated.

Several other exercise-induced mechanisms in the hypothalamus could contribute more indirectly to energy balance and healthy metabolic function. Excessive lipid mass can result in increased endoplasmic reticulum stress that inhibits liver insulin actions, and is a molecular mechanism that contributes to the onset of type two diabetes [69] and increases the risk of progression in Alzheimer’s disease [7,8]. In contrast to expectations, three weeks of VWR in mice fed high-fat diets increased levels of endoplasmic reticulum stress (ERS) [70]. The ERS marker ATFF6 was increased for high runners and low runners, while eIF2α was increased in high runners only [70]. The metabolic effects of exercise therefore appear not be related to exercise-induced reductions in ERS. However, it is also possible that physiological mechanisms involved with ERS due to exercise differ from those resulting from a high fat diet, and further research could aid in clarifying this issue. Additional effects of exercise are evident in mitochondria, that produce more than 90% of cellular energy [71] required for undertaking cellular functions (for further review see [72]). Moreover, in 8 week old mice, eight weeks of forced treadmill running (6 days/week at 25metres/min with 5% incline) significantly increases the mitochondrial DNA (mtDNA) copy number relative to nuclear DNA in the hypothalamus [73]. A limitation of this study is that there is no examination of comparisons with older mice or the use of VWR to determine potential differences evident due to these factors. Nonetheless, a relatively demanding treadmill running protocol can contribute to improving hypothalamic cellular energy dysfunction.

### Exercise-induced cardiovascular system changes

It is now widely recognised that chronic regular exercise has an important role in cardiovascular health [74] although the neurophysiological mechanisms responsible for cardiovascular function are less well understood. The medullary nucleus tractus solitarii (NTS) is thought to have a fundamental role in coordinating complex adaptations to exercise through communication with the hypothalamus [75]. Chronic exercise related adaptations to the CNS kallikrein-kinin system might contribute to this function. The kallikrein-kinin system generates peptides involved in sodium regulation, blood pressure, and inflammation [76]. The activation of B2 kinin receptors, a mediator of the effects of kinins in this system, are involved in the modulation of cardiovascular responses to stress [77]. In hypertensive rats, as in humans, central kinin B2 receptor density is higher in several brain regions including the medullary nuclei [77]. Ten weeks of treadmill exercise at 50-70% V02max in male Wistar rats increased specific B2 receptor binding sites in the paratrigeminal nucleus and nucleus solitarii, as well asincreased receptor density in the medulla [77]. This suggests enhancement of the kallikrein-kinin system function may modulate the cardiovascular responses to exercise or stress [77]. However, treadmill running also affects mechanisms relating to autonomic function. Treadmill running for 3 months at 50-60% Vo2 max, significantly increased oxytocin mRNA levels in the commissural NTS in male normotensive rats, which was associated with increased autonomic cardiac function [78]. However, these mechanisms require investigation using VWR protocols due to potential confounding factors from forced exercise. Nonetheless, exercise-induced effects from VWR are also evident. VWR for 50 days resulted in dendritic plasticity seen as reduced dendritic intersecting per dendritic field in exercised rats compared to sedentary controls in the NTS, posterior hypothalamus, periaquaductal gray, rostral ventrolateral medulla and nucleus cuneatus [79]. Of note is that the dendritic plasticity was related to peak physical performance [79]. These results are pertinent, as it has been suggested that greater dendritic branching (in the RVLM) may contribute to greater sensitivity in these neurons that mediate excitatory responses, thereby contributing to the pathogenesis of cardiovascular disease [80].

Regulation of the autonomic nervous system is also critical to central cardiovascular function, and forced exercise may contribute to the modulation of these systems. In rodents, forced protocols have demonstrated modulation of central cardiovascular neural controls, leading to modified resting cardiovascular parameters such as mean arterial pressure, and heart rate, and reduced sympathetic nervous system (SNS) activity [81-85]. These adaptations occurred through enhanced basal GABAergic function via increased neural nitric oxide synthase (nNOS), that inhibits sympathetic outflow from the paraventricular nucleus (PVN)); GAD67 (which converts glutamine into the inhibitory neurotransmitter GABA); and gephyrin (a component of inhibitory synapses in the anterior and posterior hypothalamus) [81]. Nevertheless, the use of voluntary running methods to eliminate the potential for physiological (psychological stress related) confounding factors is likely to provide more sound and translatable results.

### HPA axis adaptations with exercise

Hypothalamic-pituitary adrenal axis (HPA) activation occurs with both psychological and physiological stressors. Excitatory signals from the amygdala, PFC and hippocampus to the PVN of the hypothalamus stimulate the release of CRH. This thenactivates the secretion of adrenocorticotropic hormone (ACTH) from the pituitary into circulation, resulting in the release of glucocorticoids (GCs) (cortisol in humans and corticosterone in rodents) from the adrenal cortex [86]. GCs then modulate and control the stress response, exerting a diverse range of effects on a wide variety of physiological systems including metabolism and immunity. Moreover, GCs, via binding to the glucocorticoid receptor (GR) inhibit the further release of CRH, thereby switching off the release of further GCs [87]. Dysfunction of the HPA axis in patients with major depression is one of the most consistent findings in biological psychiatry. Patients with depression have increased plasma and CSF concentrations of GCs, an exaggerated GC response to ACTH, and also appear to have dysregulation of the inhibitory feedback of GCs [88]. HPA axis responses are therefore a critical dimension of the treatment of these conditions.

The HPA axis response to voluntary exercise occurs as outlined above. It is a normal adaptive mechanism in response to the increased energy requirements of peripheral tissues, and is a physiological stressor without the psychological stress of fear [86] (unless exercise is forced when this potential is present). Chronic VWR has demonstrated effects on HPA axis parameters in rodents, including increased size and mass of the right adrenal medulla, adaptive changes in ACTH levels [89], and the normalisation of GC levels [90,91]. There is also evidence that VWR can attenuate the HPA axis response to psychological stressors. VWR has been demonstrated to attenuate rises in plasma ACTH arising from foot-shock and cage-switch stressors [92]. Moreover, 6 weeks of VWR in male Sprague–Dawley rats attenuated HPA axis responses to low intensity stressors, such as exposure to a novel environment, 85 decibel (dB) noise, and this was more successful than 1 or 3 weeks of VWR [93]. These results are consistent with other work demonstrating greater habituation to noise stressors with VWR [94,95]. It should be noted though that other studies have found no changes in plasma ACTH with VWR after repeated foot-shock [92]. These inconsistencies could be due to the varied stressors involved, however, additional research would clarify this hypothesis.

In the hypothalamus, research has investigated VWR induced adaptations in HPA axis parameters including CRH mRNA, *c-fos*, arginine vasopressin, and CRH receptor 1 mRNA [93,96]. Six weeks of *ad libitum* wheel running reduced CRH mRNA in the hypothalamus in the context of repeated noise stressors; and both *ad libitum* and intermittent (24 hours out of 72) access to VWR resulted in a significant reduction of *c-fos* expression in the paraventricular nucleus of the hypothalamus [93]. No changes however, were found in arginine vasopressin or CRH receptor 1 mRNA in the paraventricular nucleus [96]. Reduced *c-fos* expression in the PVN with voluntary and intermittent wheel running could suggest attenuated activation of the PVN neurons that may contribute to reduced excitatory input from the PVN to the pituitary, potentially resulting in a reduction in the release of ACTH. Moreover, it is encouraging that relatively reduced (intermittent) access to VWR can have positive effects on PVN *c-fos* expression.

The findings of exercise-induced changes in central parameters of the HPA axis may be obscured when forced exercise methods such as treadmill running are used. For example, one study investigating the effects of incrementally increasing forced swimming for 6 weeks found decreases in hypothalamic glucocorticoid receptor mRNA (p < 0.01) from weeks 2 to 4 that remained unchanged to week 6, with transient increases in CRH mRNA from week 2–4 in the PVN [97]. In addition, 19 days of treadmill exercise was also found to modulate chronic corticosterone administration induced HPA axis hypoactivity [98]. It should be noted that the potential stress involved in forced treadmill training, which is in addition to the physiological effects of exercise stress, might confound these results. Thus, the inclusion of a voluntary exercise group as a control in these experiments would aid in elucidating the direct physiological effects of exercise versus those caused by psychological stress.

HPA axis activation in response to exercise occurs in both male and female rodents, but in females this varies in relation to the oestrus cycle [86,99]. To the authors knowledge there were no papers returned from our searches that investigated differences between male and female chronic VWR induced hypothalamic markers of HPA activation. Factors such as CRH or CRH receptor adaptations with VWR, the acute effects of VWR on female hypothalamic HPA activation at different stages of the oestrus cycle, and the effects of exercise on these factors in the context of stress remain unexamined. These are highly noteworthy limitations of the literature at present, given that the prevalence of depression has consistently been demonstrated to be higher in females than males in humans [100,101], and that 80% of clinical depression is preceded by chronic psychological stress [102-104]. Furthermore, these findings suggest that the controllability of exercise, its frequency, and duration, and the sex of the animal undertaking exercise are all potential factors involved in moderating the effects of exercise on hypothalamic input into the HPA axis. The perception of stress during forced exercise is likely to vary between individuals, whether human or rodent, and add to the physiological stress of exercise. Speculatively speaking, this additional stress might constitute a mechanism whereby forced exercise - or psychologically stressful exercise - could exacerbate clinical symptoms of stress, and stress related conditions such as stress induced depression. The VWR induced effects on hypothalamic HPA axis function in female mice in particular, is a gap in the literature urgently requiring examination by future research.

## Exercise-induced adaptations in the basal ganglia

The basal ganglia includes the striatum, comprised of the putamen, caudate nucleus, and nucleus accumbens, as well as the globus pallidus, the subthalamic nucleus and substantia nigra [105]. These nuclei, and the putamen in particular, have roles in the control of muscle tone control and movement due to the input received from the somatosensory and motor cortices, with output tothe motor areas of the cortex [105]. Dysfunction in these regions can lead to bradykinesia and tremors that can severely limit activities of daily living as occurs in Parkinson’s disease.

Clinical studies investigating the effects of exercise for the treatment of Parkinson’s disease have found task based exercise can aid in improving functional mobility [106], although the mechanisms involved are not well understood. Nevertheless, basic science studies investigating the mechanisms of exercise in the basal ganglia demonstrate changes in oxidative stress markers and antioxidant equilibrium. Moderate treadmill running for 8 weeks increases levels of rodent striatal tyrosine hydroxylase (TH) (an enzyme that catalyses L-tyrosine into dihydroxyphenylalanine or L-DOPA, a dopamine precursor) and returns α-synuclein phosphorylation (a protein involved in Lewy body conditions) to close to normal levels [107]. However, this is in contrast to another study, which noted no changes in TH in the substantia nigra pars compacta with treadmill exercise [108]. It is possible that the forced component of treadmill exercise altered and the mechanisms involved and confounded outcomes, and this highlights the importance of using voluntary exercise methods. TH levels are important because dopamine depletion is a central factor in the aetiology of Parkinson’s disease [107]. The potential for TH increases with VWR exercise requires further investigation because it may increase the availability of TH for synthesis into L-DOPA. This has important implications for translation to clinical treatment of Parkinson’s disease in humans.

Mixed results are evident about levels of oxidative stress in the basal ganglia in response to exercise. Striatal levels of thiobarbituric acid reactive substances (TBARS), that are involved in cellular oxidative damage, were reduced by treadmill running at 13–17 metres/minute for 3 or 4 days a week [107], but not from exercise for 5 days/week for 8 weeks at 10 m/min, 15 m/min, or 20 m/min [109]. It is noteworthy that treadmill running has been reported to significantly reduce other markers of oxidative damage, such as carbonyl content [107,110], while the antioxidant enzyme superoxidase dismutase (SOD) (an enzyme that catalyses the cellular antioxidant mechanism of superoxide into oxygen and hydrogen peroxide) was found to increase [107]. However, these results also require confirmation with studies using voluntary methods.

Exercise also induces alterations in striatal brain derived neurotrophic factor (BDNF). BDNF is thought to be important for the survival of dopaminergic neurons in the striatum. Thus a lack of BDNF in the striatum has implications for dopamine transmission, as well asfor dopamine deficiency related mobility dysfunction conditions such as Parkinson’s disease [11,111]. Striatal BDNF mRNA levels are increased significantly (p = 0.01) with 3 weeks of VWR [112]. Moderate to high intensity downhill treadmill running also increases BDNF protein (p = 0.001) [113], although 18 weeks of level treadmill running does not appear to increase BDNF increase [110]. Interestingly, chronic treadmill running also normalises levels of striatal glial fibrillary acidic protein (GFAP) in mouse models of Parkinson’s disease [108,114] suggesting that reductions in markers of pathology may also be possible in humans with this condition.

Conversely, high intensity exercise may have detrimental effects in this region. In the striatum, high intensity treadmill exercise disrupts ERK ½ and CREB pathways. This was associated with impairments in implicit memory [115]. Similarly, six months of VWR in female Long-Evans hooded rats significantly increased COX activity in the dorsolateral caudate putamen (p < 0.01) [116]. These findings are consistent with recent systematic review findings suggesting that higher intensity exercise may be detrimental to anti-oxidative capacity in humans [13]. However, high intensity treadmill exercise also increases striatal D2 receptor levels, prevents dopamine transporter protein down regulation [117] and reduces pathological glutamatergic neuroexciteability in the striatum [118]. In addition, moderate chronic treadmill running increases striatal nitrergic nitric oxide synthase (NOS) reactivity suggesting up-regulation of the striatal nitrergic system [119]. This is noteworthy because NOS are signalling molecules implicated in synaptic plasticity that are diminished in degenerative diseases. Overall then, clarity about the benefits versus risks of high intensity exercise in the striatum remains unresolved.

## Limitations of the review

To the author’s knowledge, this review constitutes the first brain region specific examination of the neurobiological effects of exercise. Moreover, the review has focussed on CNS functions that become dysfunctional in prevalent conditions such as depression, Parkinson’s disease and Alzheimer’s disease, factors that are therefore highly pertinent in the current context of globally ageing populations and projected increases in these conditions. However, although this region specific approach provides a novel and worthwhile insight into exercise neuroscience, it does involve some limitations. The examination of other important brain regions, including the limbic system and its interactions on the CNS functions presented herein are complex, and require in depth investigation. Unfortunately, limitations of space preclude such investigations in the present review. Another possible limitation of this review may be that the inclusion of only English published articles could contribute to some selection bias in the results of the review.

## Conclusions

Considerable research has now focussed on the effects of exercise in clinical populations and higher brain regions such as the hippocampus, resulting in greater knowledge about how exercise might support cognitive functioning. However, there appears to be relatively little literature on the effects of exercise on critical centrally mediated mechanisms that involve the functioning of more primitive brain regions.

Nevertheless, this paper has reviewed murine studies examining the effects of exercise on the brain stem, hypothalamus, and basal ganglia that constitute basic CNS functions that are critical for health. Important functions of these regions include the circadian clock; energy balance and metabolism; responses to stress and HPA axis functioning; and the maintenance of normal mobility. The functioning of these systems within normal physiological ranges promotes health. Importantly, the dysfunction of these systems is increasingly considered involved in the pathogenesis of a range of prevalent conditions such as depression, Alzheimer’s disease, and Parkinson’s disease.

**Figure 2:**
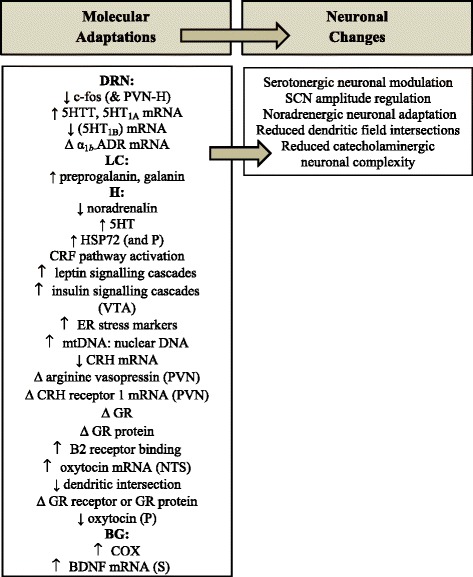
Effects of voluntary exercise in the brain stem, hypothalamus, and basal ganglia. Legend: Δ = no change; BDNF mRNA = brain derived neurotrophic factor mRNA; c-fos = protein induced acutely by several factors including cytokines; COX = cytochrome oxidase, an indicator of brain regional functional activity; CRF = corticotropin releasing factor/hormone; 5HT = serotonin; 5HTT = serotonin transporter; 5HT1A mRNA = serotonin receptor 1A mRNA; 5HT1B mRNA = serotonin receptor 1B mRNA; Δ α1b-ADR mRNA = α1b-adrenergic receptor (α1b-ADR) mRNA; α-synuclein = precursor protein of amyloid; DRN = dorsal raphe nucleus; ER = endoplasmic reticulum; galanin = a regulatory peptide cleaved from preprogalanin; GR = glucocorticoid receptor; H = hypothalamus; HSP72 = heat shock protein 72; mtDNA: nuclear DNA = mitochondrial DNA to nuclear DNA ratio; NOS = nitric oxide synthase; NTS = nucleus tractus solitarii; P = pituitary; preprogalanin = a precursor of galanin; PVN = paraventricular nucleus; BG = basal ganglia; S = striatum; VTA = ventral tegmental area.

The findings reviewed indicate that exercise induces numerous molecular and neuronal adaptations in the brain stem, hypothalamus and basal ganglia. However, a proportion of this work involves forced methods that may differentially affect neurophysiological mechanisms due to the potential for physiological cascades in response to the psychological stress involved in forced exercise. This can confound results [12] leading to misleading findings. Running at intensities greater than are physiologically established by the animal could have adverse effects in some instances [115], and has the added problem of difficulty in the translation to human contexts. In contrast, studies using voluntary wheel running methods have identified a range of regional exercise-induced molecular neurophysiological mechanisms that may contribute to desirable changes in brain region specific functions (see Figure 2).

Voluntary exercise-induced mechanisms mediating stress responsivity in the DRN include serotonergic and adrenergic modulation [32-35] and preprogalanin and galanin in the LC (noradrenergic modulation) [37,40-42]. Hypothalamic metabolic parameters altered by exercise include CRF and leptin signalling modulation [59,60], and changes in food intake [62,63] and markers of MetS [62]. Also in the hypothalamus, exercise-induced increases in B2 receptor bonding sites and dendritic field reductions [77,79] may contribute to altered cardiovascular function. Exercise-induced changes in HPA axis functioning in the hypothalamus appear to be mediated by reduced c-fos expression in the context of exposure to stressors, reduced pituitary oxytocin, and increased HSP72 [52,93]. Finally, in the basal ganglia, voluntary wheel running increases COX activity in the putamen and elevates BDNF mRNA in the striatum [112,116].

Attention to a number of methodological issues by future research will advance the field of exercise neuroscience. First, the forced exercise related findings from all brain regions require replication and confirmation with voluntary wheel running studies. Second, if forced methods are continued, consensus ought to be sought and agreed upon regarding standardised intensities to enable comparable research in the field and the translation to clinical trials. Third, adequately powered studies inclusive of female animals are urgently required to address the gap in the literature about the regional neurobiology of exercise in females. Fourth, future investigated parameters would benefit from the examination of exercise at different ages, to ascertain the effects of exercise throughout the lifespan. This is particularly salient for parameters pertaining to age related conditions such as Parkinson’s disease and Alzheimer’s disease. By incorporating these considerations into future studies, considerable opportunities to advance exercise neuroscience are available that will result in better understanding of regional brain dysfunctions involved in the aetiology and progression of conditions such as depression, Alzheimer’s disease, Parkinson’s disease, and many others.

## Abbreviations

CNS: Central nervous system
HPA: Hypothalamic pituitary axis
VWR: Voluntary wheel running
DRN: Dorsal raphe nucleus
RVLM: Rostral ventrolateral medulla
GABA: Gamma-aminobutyric acid
LC: Locus coeruleus
SCN: Suprachiasmic nucleus
HSP72: Heat shock protein 72
MetS: Metabolic syndrome
CRF: Corticotropin releasing factor
IRS2: Insulin receptor 2
VTA: Ventral tegmental area
NTS: Nucleus tractus solitarii
ERS: Endoplasmic reticulum stress
SNS: Sympathetic nervous system
nNOS: Neural nitric oxide synthase
PVN: Paraventricular nucleus
ACTH: Adrenocorticotrophic hormone
GCs: Glucocorticoids
GR: Glucocorticoid receptor
TH: Tyrosine hydroxylase
TBARS: Thiobarbituric acid reactive substances
SOD: Superoxide dismutase
BDNF: Brain derived neurotrophic factor
NOS: Nitrergic nitric oxide synthase
GFAP: Gial fibrillary acidic protein
MPTP: 1-methyl-4-phenyl-1,2,3,6,-tetrahydropyridine
